# Severe peripheral arterial diseases in hemodialysis patient: A case report

**DOI:** 10.1097/MD.0000000000018760

**Published:** 2020-01-24

**Authors:** Jiali Li, Mingming Yan, Jiao Qin, Jun Liu, Rui Wen

**Affiliations:** a Department of Nephrology, University of South China affiliated Changsha Central Hospital; b Department of Orthopaedic Surgery, The Second Xiangya Hospital, Central South University, Hunan Province, China.

**Keywords:** hemodialysis, mesenteric artery disease, percutaneous transluminal angioplasty, peripheral arterial diseases

## Abstract

**Rationale:**

Peripheral arterial diseases (PADs) is defined as a systemic arterial disorders involving the lower extremity arteries, iliac, and carotid, which is developed more common in patients with chronic kidney disease (CKD) than individual with normal renal function. Concurrence of mesenteric artery disease and lower extremity artery disease (LEAD) is rare. The presence of PADs in patients receiving hemodialysis leads to a dramatic increase in risk of cardiovascular mortality. However, the early diagnosis of PADs in patient with CKD remains a challenge to nephrologists, which adds an adverse effect on prognosis.

**Patient concerns:**

A 48-year-old man received regular hemodialysis due to end-stage renal failure caused by type 2 diabetes mellitus (T2DM) for 7 years, who was admitted into hospital for acute, severe rest pain of the right lower extremity at the first time. The computed tomography angiography showed severe, diffuse stenosis of the distal third of femoral artery. After discharged, he was readmitted into hospital for abdominal pain and the recurred right lower limb pain. A diagnostic angiography confirmed the initial occlusion of superior mesenteric artery, severe obstruction of the distal segment of femoral artery and diffuse, irregular stenosis of arteria peronea and arteria tibialis posterior.

**Diagnosis:**

The patient was diagnosed as PADs including LEAD and mesenteric artery disease.

**Interventions:**

The percutaneous transulminal angioplasty (PTA) combined with antiplatelet therapy and beraprost were performed. Moreover, the cinacalcet and lanthanum carbonate were prescribed to control calcium-phosphorus- parathyroid hormone metabolism.

**Outcomes:**

The patient was free of abdominal pain and partly relieved from the ache of lower limb after PTA. However, he finally succumbed to acute myocardial infarction.

**Lessons:**

The incidence of PADs is higher in dialysis patients due to a unique set of biochemical and endocrine abnormalities. As there is a high uremic status and PADs burden in patients with hemodialysis, the short term risk of cardiovascular disesase mortality markedly increases. There is a need for nephrologists and cardiovascular physicians to identify these patients and then provide early and proper treatment.

## 1 Introduction

Peripheral arterial diseases (PADs) encompass all arterial diseases including carotid artery disease, mesenteric artery disease, renal artery disease and lower extremity artery disease (LEAD), other than coronary arteries and the aorta.^[1]^ This should be clearly distinguished from the traditional definition of PAD, which is often described for LEAD.

LEAD caused by atherosclerotic occlusion of the arteries to the lower limb is common in dialysis patients, while mesenteric artery disease is rare. The term ‘mesenteric artery disease’ was first put forwarded into 2017 ESC guidelines on diagnosis and treatment of PADs, which resulted from acute and chronic occlusion or stenosis of the mesenteric arteries, especially the superior mesenteric arteries (SMA).^[1]^ Based on our knowledge, there are few reports about the patient with concurrent mesenteric artery disease and LEAD. In this article, we reported a middle-aged male man on regular hemodialysis developed both mesenteric artery disease and LEAD. Satisfied outcome was obtained after percutaneous transulminal angioplasty (PTA) and medical therapy, but he eventually succumbed to acute myocardial infarction (AMI).

## 2 Case report

A 48-year-old man with a history of end-stage renal failure (ESRD) caused by type 2 diabetes mellitus had been hemodialyzed for 4 hour, 5 times per 2 weeks for 7 years. He suffered from the severe, refractory arterial hypertension when he was diagnosed with cardiovascular disease (CVD). However, he occasionally began to experience interdialytic hypotension in recent 1 year. In addition, he had quit smoking for 10 years.

**Figure 1:**
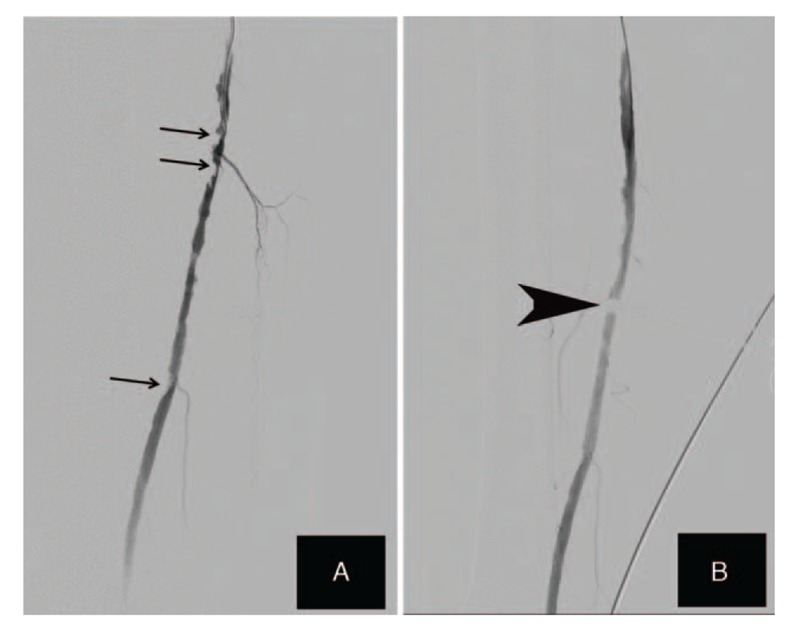
(A) Severe and diffuse stenosis of the femoral artery. (B) Stenosis reduced markedly and good blood flow in the femoral artery. Arrows indicate stenosis of femoral artery before PTA, the arrow head indicates tweezers after PTA. PTA = percutaneous transluminal angioplasty.

He was admitted into hospital for acute, moderate rest pain of the right lower extremity in April 2017. The physical examination at admission showed a high blood pressure (160/72 mm Hg) and mild weak pulses of the dorsalis pedis artery in the right foot. The ankle-brachial index (ABI) for left limb was 0.84 and the measurement of ankle-brachial index of right limb was not available due to arteriovenous fistula in right forearm. Laboratory tests revealed an elevated serum creatinine level of 867 umol/L, serum phosphate (1.5 mmol/L) and i parathyroid hormone (PTH) (772.7 umol/L), but normal calcium (2.14 umol/L). Admission angiography showed severe, diffuse stenosis of the distal third of femoral artery (Fig. 1a). The patient was diagnosed as LEAD and treated by PTA for revascularization. The contralateral approach was performed under local anesthesia. The 5-F sheath was inserted in the left femoral artery and heparin (4000IU) administered to intra-arterially. After that, guide wire (0.014 inch) was used to deliver balloon to the lesions. The diffuse stenotic lesions in the distal third of femoral artery were dilated by inflating the balloon catherter to 5 mm in diameter at 6 atm for 180 second. Angiography after PTA revealed a markedly decrease in length of severe, diffuse stenosis femoral artery and good blood flow obtained (Fig. 1b). After the procedure, the antiplatelet agents aspirin (200 mg per every other day) and clopigrel (75 mg per day) were administered orally.

The patient was discharged after 7 days in good condition. However, the antiplatelet drugs were not taken regularly at home due to the discomfort of gastrointestinal. In June, he had intermittent postprandial abdominal pain for 1 month, which led to fear of eating and progressive weight loss of about 5 kilograms. He was readmitted for severe abdominal pain, accompanied by nausea, vomiting, and poor general status. Clinical examination showed a high blood pressure (172/66 mm Hg) and tachycardia (106 beats per minute), abdominal tenderness without guarding or other peritoneal signs. The right limb was cold and pale, and only femoral pulse was palpable. Laboratory tests revealed normal leukocytosis (7.2 × 10^9^/L), elevated serum creatinine (622.5 umol/L), dramatically elevated serum phosphate (2.26 mmol/L), serum calcium (2.56 mmol/L) and iPTH (1152.4 pg/mL). The computed tomography angiography performed in last 2 month revealed the irregular calcific stenosis of initial segment of SMA. Considering the discrepancy between the severity of symptoms and an almost normal clinical examination, we decided to perform PTA in order to restore the bowel viable. Moreover, the recurrence of symptoms in right lower limb made us examine the right femoral artery again.

**Figure 2:**
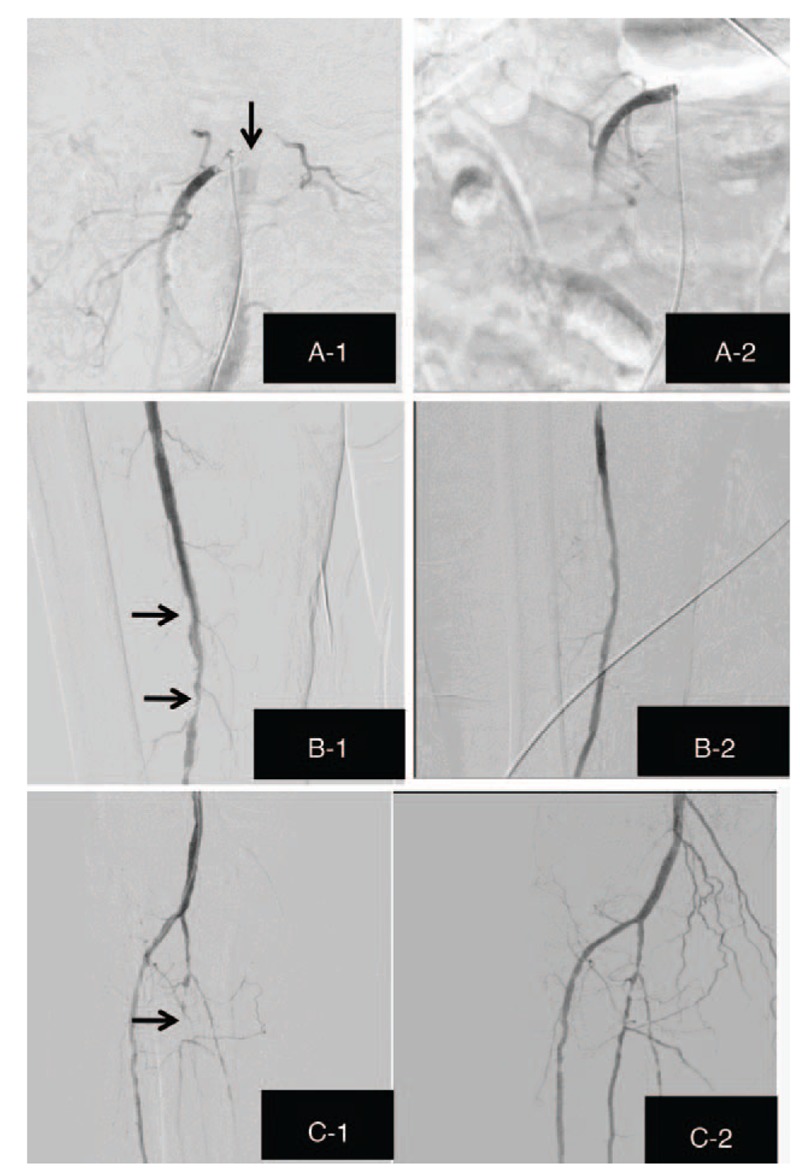
Digital substraction angiograms of the initial segment of SMA. Occlusion of initial segment of the SMA before PTA (A-1). No significant residual stenosis of initial segment of SMA after PTA (A-2); Digital substraction angiograms of femoral artery. Severe obstruction of the distal segment of femoral artery (B-1). Good blood flow in femoral artery (B-2); (C) Digital substraction angiograms of arteria peronea and arteria tibialis. Diffuse, irregular stenosis of arteria peronea and arteria tibialis posterior and occlusion of proximal segment of arteria peronea (C-1). A partial stenosis of arteria peronea and arteria tibialis (C-2). PTA = percutaneous transluminal angioplasty, SMA = superior mesenteric artery.

After a diagnostic angiography confirmed the initial occlusion of the SMA (Fig. 2A-1), we chose a femoral approach using a 6F sheath and intra-arterial catheter thrombolysis with heparin (3000IU). The lesion was crossed with some difficulty when using a 0.014-inch guidewire, so we chose 6F guiding catheter through the obstruction again. After crossing, the occlusive initial segment was predilated with a coronary 4.5/12 mm balloon at 3 atm for 120 second. After a second inflation with the 6/60 mm balloon catheter protruded in SMA, an acceptable outcome with no significant residual stenosis and complete pain relief was achieved (Fig. 2A-2).

Digital subtraction angiography of the lower limbs revealed severe obstruction of the distal segment of femoral artery (Fig. 2B-1), diffuse, irregular stenosis of arteria peronea and arteria tibialis posterior (Fig. 2C-1). The occlusive lesion in femoral artery was dilated by inflating a 5/15 mm balloon catheter with urokinase (10000IU) administered intra-arterially. A 2.5/12 mm balloon was then inflated to further dilate the proximal segment of arteria peronea and arteria tibialis posterior. Images after PTA were shown in Figure 2B-2 and C-2. After the PTA procedures, the patient's pain was relieved and aspirin (100 mg per day), Beraprost sodium (40 ug three times per day) and clopigrel (75 mg per day) were administered orally. Moreover, the cinacalcet and lanthanum carbonate were also prescribed to alleviate the elevated serum phosphate and iPTH

During the 1 month after the acute event, the patient was asymptomatic in abdomen. However, he gradually presented with an intractable decubitus ulcer on the dorsum of right foot and dry gangrene of the right fifth toe 1 month later. Unfortunately, his overall condition deteriorated and he died of AMI.

## 3 Discussion

The incidence of mesenteric artery disease combined with LEAD in hemodialysis patient is rare, which makes it difficult for nephrologists to establish the diagnosis at its early stage. Patients receiving hemodialysis have higher morbidity rate of PADs compared to general population due to the unique set of biochemical and endocrine abnormality. First, the chronic uremic state related to systemic inflammation in hemodialysis patients is prevalent.^[2,3]^ Hypercoagulation, oxidative stress injury and some inflammatory factors participate in the development of atherogenesis. Second, hyperphosphatemia has been demonstrated to predict PAD events in a small case control study.^[4,5]^ Third, secondary hyperparathyroidism (SHPT) is one of the major problems among long-term dialysis patients. Previous studies showed SHPT could accelerate atherosclerotic processes by targeting endothelial cells^[6]^ and induce elevation of serum calcium and phosphorus. Therefore, these metabolic changes play an essential role in the pathological process of vascular calcification in intima and medial layer of muscle, which might contribute to the PADs development in ESRD.^[7,8]^

In general, the therapeutic approach to CKD patients with PADs should pay attention to the following:

(1) To deal with the symptoms of the local lesion. The revascularization is often performed to restore the bowel viability, alleviate the abdominal pain in mesenteric artery disease and improve limb symptoms or limb salvage in LEAD. The endovascular therapy and open surgery are 2 main popular surgical options for revascularization. The endovascular therapy is more feasible and effective than open surgery for dialysis patients due to its merit of less invasive and fewer complication and postoperative mortality^[9]^;
(2) To prevent the increased risk of cardiovascular (CV) event. The general management of CV event prevention was utmost, which included medical therapy as well as lifestyle changes such as smoking cessation, healthy diet, weight loss and regular physical exercise. It has been advocated that smoking cessation is regarded as the keystone of nonpharmacological management for PADs. The principal medical therapy is composed of antihypertensive, lipid-lowering, and antithrombotic drugs; and
(3) To control calcium-phosphorus-PTH metabolism. Due to the prevalent imbalance of PTH and phosphate level in patients receiving hemodialysis, they have higher risks to develop PADs than general population without CKD. Accordingly, the therapeutic approaches should include parathyroidectomy, cinacalcet and none-calcium based phosphate binders to control calcium-phosphorus-PTH metabolism in hemodialysis patients.

PADs are associated with increased risks of CV mortality in patients on hemodialysis. Data from a meta-analysis showed that a significantly higher CV mortality in hemodialysis patients with PADs (risk ratio: 2.99, 95% confidence interval: 1.65–5.36).^[10]^ In present study, the patient died of AMI nearly 1 year after he suffered from PADs. The severe CV event may be caused by the following factors. PADs resulted from the similar risk factors of CV disease such as male sex, diabetes, hypertension, and smoking.^[11]^ In addition, as PADs and CV disease are all usually secondary to atherosclerosis, the narrow and obstruction lesions caused by atheroscleroticplaque may exist together in peripheral arteries and coronary artery.^[10]^ Moreover, due to a high uremic status and PADs burden in patients on dialysis, the short term risk of CV mortality markedly increases.^[12]^ Therefore, it is of great necessity for nephrologists and cardiovascular physicians to identify these patients and provide early and proper treatment.

## 4 Conclusion

Dialysis patients have higher PADs prevalence rates due to a unique set of biochemical and endocrine abnormalities. Most patients are present with LEAD while mesenteric artery disease is rare. Endovascular revascularization is a feasible and effective theraputic approach in PADs patients. When PADs and dialysis are present together, the mortality caused by CVD disease is extremely high.

## Author contributions

**Investigation:** Jun Liu.

**Resources:** Mingming Yan, Jiao Qin.

**Writing – original draft:** Jiali Li.

**Writing – review and editing:** Rui Wen.
